# Rhino-orbital-cerebral mucormycosis with Klebsiella and MRSA co-infection in a diabetic patient: a case report

**DOI:** 10.1097/MS9.0000000000002416

**Published:** 2024-08-02

**Authors:** Ram Chandra Subedi, Ayush Adhikari, Shekhar Gurung, Pinky Jha, Subi Acharya, Tibbin Kumar Shiwakoti, Bhuwan Bhatta, Naresh Kharbuja, Barada Lamichhane, Raju Paudel, Saroj Kumar Jha

**Affiliations:** a Department of Neurology, Grande International Hospital; b Department of Anesthesia and Critical Care, Tribhuvan University Teaching Hospital; c Department of Emergency Medicine, Chattarapati Free Health Clinic Community Hospital; d Nepalese Army Institute of Health Sciences; e Department of Internal Medicine, Kantipur Hospital, Kathmandu; f Department of Pediatrics, Patan Academy of Health Sciences, Lalitpur; g Department of General Practice and Emergency Medicine, Karnali Academy of Health Sciences, Karnali; h Department of Internal Medicine, Gandaki Medical College, Pokhara; i Department of Internal Medicine, Gajendra Narayan Singh Hospital, Rajbiraj, Nepal

**Keywords:** case report, diabetes, invasive fungal infection, mucormycosis, Rhino-orbito-cerebral

## Abstract

**Introduction and importance:**

Rhino-orbital-cerebral mucormycosis is an opportunistic infection caused by fungus species Rhizopus and Mucor. Early recognition and aggressive management is crucial for favorable outcomes. A delay in diagnosis and treatment is fatal.

**Case presentation:**

A 32-year-old female presented with high-grade fever, right-sided facial deviation associated with facial swelling, and inability to move her left eye for 10 days. Biopsy from the left nasal cavity showed fibrinoid material, edema, and sheets of neutrophilic infiltrate while KOH preparation of nasal scrapping showed aseptate hyphae with obtuse-angled branching. Amphotericin B, oral posaconazole, and antibiotics were started with exploration and debridement of the affected tissue. The patient recovered well and was discharged.

**Discussion:**

Immunocompromised people are primarily affected by mucormycosis, a serious fungal illness. Inhaling fungal spores, especially those of the Rhizopus and Mucor species, is the usual cause. Rhinocerebral mucormycosis (ROCM), the most common type, increased during COVID-19 pandemic, frequently as a result of hyperglycemia brought on by steroids. Angioinvasion and tissue necrosis are pathogenesis-related processes that are made worse by diabetes and the overuse of glucocorticoids. Histopathology, culture, and imaging are used in the diagnosis. Surgery and antifungal drugs like Amphotericin B are used in treatment. Early intervention and interdisciplinary care, including hyperbaric oxygen therapy, are critical for survival. Results deteriorate with postponed therapy, underscoring the urgency of prompt action.

**Conclusion:**

Mucormycosis should be kept in mind while formulating differential diagnosis of infective pathology in immunocompromised patients. Early diagnosis and treatment are important in improving patient prognosis in rhino-orbital-cerebral mucormycosis.

## Introduction

Highlights

- Mucormycosis, one of the opportunistic infections caused by Rhizopus and Mucor species, is often considered highly fatal and rapidly progressive.
- Mucormycosis can be present in different forms such as cutaneous, Pulmonary, rhino-orbital-cerebral, gastrointestinal, disseminated, and some rare forms such as endocarditis and osteomyelitis.
- Among these forms of mucormycosis, the Rhino-orbital-cerebral mucormycosis (ROCM) is most commonly seen in patients with diabetes mellitus.
- This fungus most commonly affects people with immune system impairments, such as those caused by uncontrolled diabetes, radiation, chemotherapy, etc.
- Histopathological analysis and direct microscopy are the gold standards for diagnosing mucormycosis.
- A multidisciplinary approach including medical therapy and surgical debridement is followed to treat the condition.

Mucormycosis is one of the opportunistic infections that may be acute or subacute, and it is often considered highly fatal and rapidly progressive. It is commonly caused by Rhizopus and Mucor species. The reported annual incidence of mucormycosis varies between 0.43 and 1.2 cases per million^1^. Mucormycosis is commonly associated with uncontrolled diabetes mellitus (DM) with ketoacidosis, hematological malignancies, hematopoietic and solid organ transplantation, and immunocompromised individuals^2^. The incidence has slightly increased during the COVID-19 pandemic due to steroid-induced hyperglycemia. Mucormycosis can present in any of the six clinical forms: Cutaneous, Pulmonary, rhino-orbital-cerebral, gastrointestinal, disseminated, and some rare forms such as endocarditis and osteomyelitis^3^. Among these forms of mucormycosis, the Rhino-orbital-cerebral mucormycosis (ROCM) is most commonly seen in patients with diabetes mellitus^4^.

ROCM occurs due to the inhalation of fungal spores, which reach the paranasal sinuses, from where it spreads into orbits and eventually gets into the skull and brain^4^. Patients with rhino orbital mucormycosis may present with features of sinusitis or may lead to periorbital cellulitis^3^.

The best test to confirm the diagnosis is a histopathological examination and fungal isolation, while the extent of damage can be made out by imaging modalities^3^. The mainstay of therapy is antifungal therapy and the treatment of the underlying cause. Surgical debridement is also advocated in some cases^5^. Here we report a case of ROCM in a diabetic patient who presented with facial nerve palsy.

We reported this case following the updated consensus-based Surgical Case Report (SCARE) Guidelines^6^.

## Case presentation

A 32-year-old diabetic female presented to our hospital via ambulance with complaints of right-sided facial deviation, facial swelling, and inability to move the left eye for 10 days. She also had intermittent fever with a maximum-recorded temperature of 101 F, which did not respond to medications. She subsequently developed drooping of the left eyelid with a decrease in vision and presented to our hospital. There was no history of similar illness in the past and no significant family history.

Her general and systemic examination was normal with only significant findings of loss of vision of the left eye with swelling and facial deviation to the right side. Vital signs were stable except for the raised temperature. On the background of diabetes mellitus and clinical findings, differential diagnoses considered were preseptal cellulitis, orbital cellulitis, and fungal infection of soft tissues. She was advised to be admitted for further evaluation and management.

**Figure 1:**
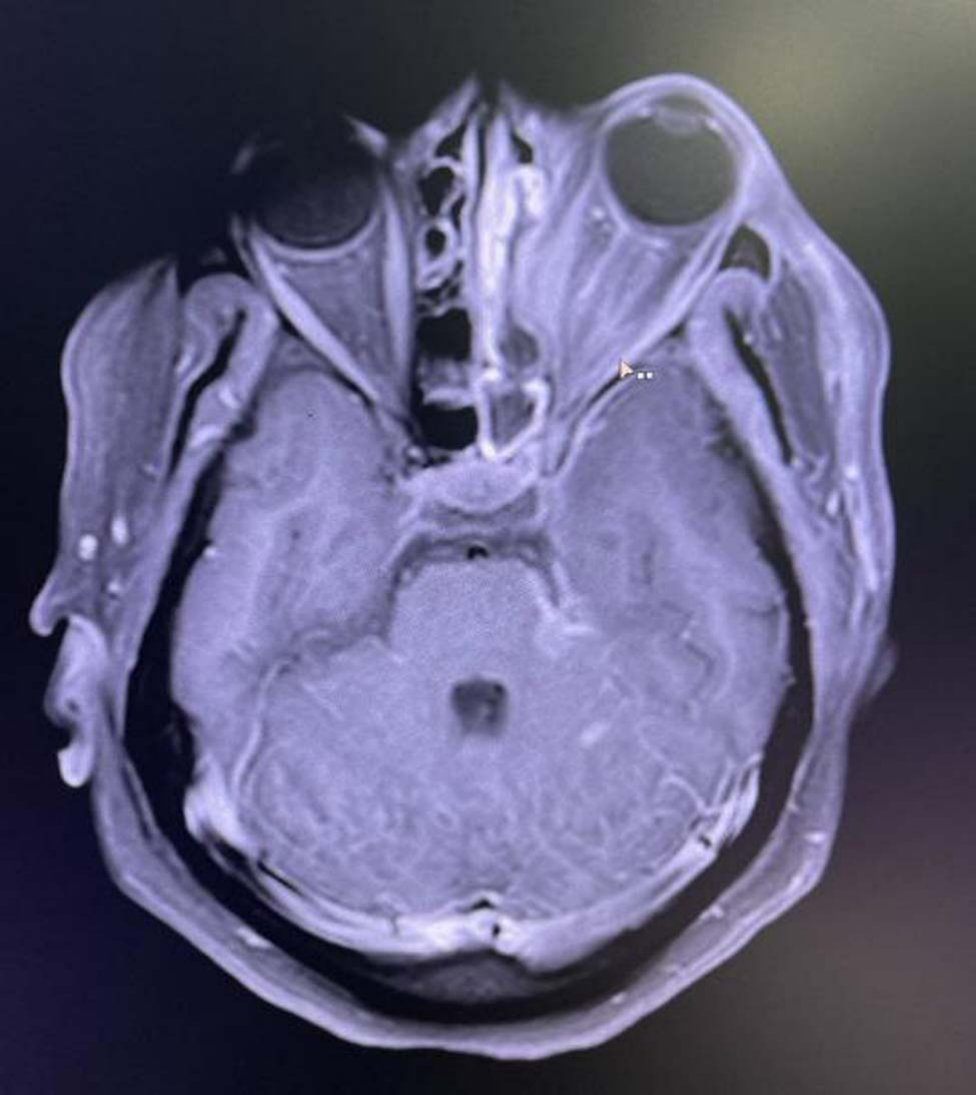
Post-contrast axial MRI showing heterogeneously enhancing soft tissue in the left ethmoidal and sphenoidal sinus with extension in the left nasal cavity. Also, enhancement was seen in extraocular muscles with mild anterior displacement of the left globe as well as in the left cerebellopontine angle along the left trigeminal nerve.

**Figure 2:**
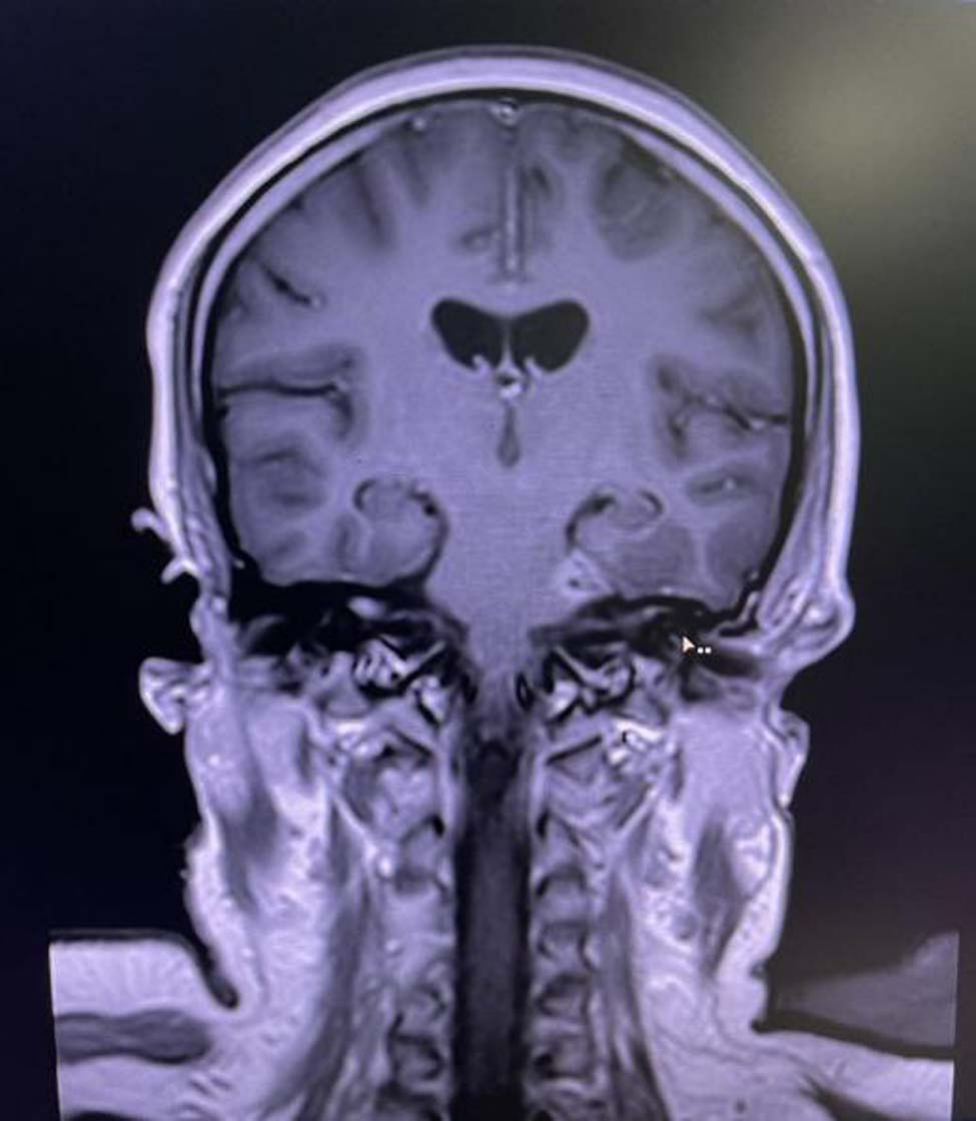
Coronal MRI showing enhancement in the left cerebellopontine angle along the trigeminal nerve.

**Figure 3:**
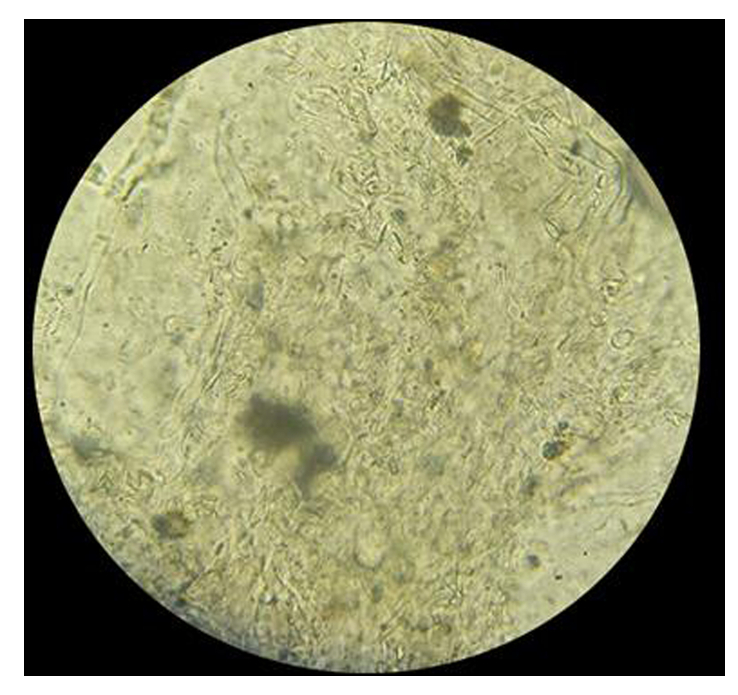
KOH staining of nasal scraping showing aseptate hyphae with obtuse angle branching.

On the day of admission, her postprandial blood glucose level was 223 mg/dl (normal: <140 mg/dl), with an HbA1C level of 14.3 (normal: ≤5.6). MRI of the head showed heterogeneously enhancing soft tissue in the left ethmoidal, maxillary, and sphenoidal sinuses with extension into the left ethmoidal, maxillary, and sphenoid sinus as well as in the left nasal cavity, with increased enhancement in the left extraocular muscles, left trigeminal nerve and axial proptosis of the left eye (Figs. 1 and 2). KOH staining of nasal scraping showed aseptate hyphae with obtuse-angled branching seen consistent with mucormycosis (Fig. 3). Thus, we diagnosed the case as rhino-orbital-cerebral mucormycosis, an invasive form of mucormycosis.

She was promptly started on broad-spectrum intravenous antibiotics—Ceftriaxone 1 g twice daily and antifungals (Amphotericin B 300 mg daily and posaconazole tablet 300 mg once daily). Due to the unavailability of a liposomal formulation, liposomal amphotericin B was changed to a non-liposomal form during the course of 28 days. Insulin and oral hypoglycemic agents were continued for diabetes mellitus. Nasopharyngolarngyoscopy with an exploration of the sinus and debridement was done multiple times during her hospital stay for one month. Aggressive endoscopic surgical debridement was considered due to the presence of necrotic tissues. The surgery was uneventful. Unfortunately, she also developed hypomagnesemia, hypokalemia, and hypocalcemia during treatment, probably due to renal tubular dysfunction induced by Amphotericin B, for which she received oral potassium supplements. During her hospital stay, she developed multiple episodes of vomiting with abdominal pain. UGI endoscopy revealed erosive gastritis, for which she was started on PPI. On the next day of admission, her Hb level was as low as 9.7 gm/dl (normal: 11.6–15.0 gm/dl), for which she received a blood transfusion. In addition, blood culture from the central venous catheter revealed Methicillin-resistant Staphylococcus aureus (MRSA) and *Klebsiella pneumoniae*. Her antibiotics were upgraded on the fourth day of admission to include Ofloxacin and Linezolid as she continued to have a fever of 102 F with a high CRP level of 109.89 mg/dl (normal: <0.3 mg/dl).

After a month in the hospital, she gradually improved and was hemodynamically stable. She was thus discharged with appropriate consultation in ENT, ophthalmology, nephrology, gastroenterology, and dermatological departments. On follow-up MRI after 9 months, there was a significant improvement in the resolution of the soft tissue of the left paranasal sinuses and left orbit, a significant reduction in the enhancement of soft tissue of the left cavernous sinus, around the left trigeminal nerve, and resolution of clival marrow edema. However, she had ophthalmoplegia of the left eye with mild optic atrophy.

## Discussion

Mucormycosis represents a group of invasive mycoses presenting as a life-threatening condition. Rhizopus oryzae and Mucor are the most often documented species in the order Mucorales of fungus^5,7^. The infection is acquired by inhalation of the fungal spores from the environment. Mucormycosis can present as cutaneous, disseminated, central nervous system, pulmonary, gastrointestinal, and other manifestations involving the musculoskeletal system, cardiovascular system, renal system, etc. Out of these, ROCM is the most prevalent and fulminant type of disease in immunocompromised individuals, as occurred in our case^7^.

This deadly fungus most commonly affects people with immune system impairments, such as those caused by uncontrolled diabetes, radiation, chemotherapy, hematologic cancers, organ transplantation, steroid therapy, patients with altered iron metabolism, patients on desferrioxamine therapy, etc^8,9^. Rhinocerebral mucormycosis, once known to be a rare occurrence, increased in incidence during the COVID-19 pandemic, especially in the second wave. Many patients of COVID-19 who were previously non-diabetic had increased their risk of developing mucormycosis owing to the development of steroid-induced hyperglycemia during the treatment^10,11^. However, our case already had diabetes mellitus, which increased her chance of developing mucormycosis.

Compared to invasive fungal infections such as aspergillosis or candidiasis, mucormycosis is a relatively uncommon infection. The affected patients’ male-to-female ratio is roughly 2:1, indicating a male predominance^5^. However, our patient was female. According to a paper by Diwakar and colleagues, ROCM was the most prevalent manifestation among the cases of mucormycosis in India, occurring in 269 (58%). The second most frequent type, cutaneous illness, affected 66 patients (14%)^9^.

The rhino-orbital-cerebral form can have a localized course (sinuses) or can extend to the orbit and/or the brain. Most frequently, the ethmoid and maxillary sinuses are the first sites where the spread begins^12^. Necrotic ulcers are seen on the nasal mucosa and turbinates during a nasal endoscopy^5^. Sinus involvement presents as nasal congestion, blood-mixed rhinorrhea, facial pain, and fever. Most frequently, ethmoidal sinus extension through the lamina papyracea results in orbital mucormycosis. Depending on the spread, the extraocular muscles are weakened, which causes gaze abnormalities^5^. Ophthalmic manifestations may include ophthalmoplegia, pain in orbit, and vision loss. A fatal side effect is the development of cerebral mucormycosis. The extension can occur directly through the frontal, ethmoid, and sphenoidal sinuses or indirectly through the ethmoidal and orbital veins that drain into the cavernous sinuses. Cavernous sinus involvement may extend seeding into one or more cranial nerves III, IV, VI, V1, and V2. Headache, confusion, discomfort, later focal neurological impairments, cranial nerve abnormalities, hemiparesis, and seizures are symptoms of cerebral involvement. In our case, fever, facial swelling, and deviation were present.

Angioinvasion, thrombosis, and tissue necrosis are all components of pathogenesis, and an activated cytokine pathway plays a significant part in the pro-inflammatory response to the fungus. In diabetic individuals, an environment high in carbohydrates encourages spore germination quickly, and in conjunction with ketoacidosis, an acidic environment promotes fungal growth^8^. The use of glucocorticoids in COVID-19 patients above the levels advised by the WHO is a risk factor for mucormycosis^13^. In contrast to ROCM, however, excessive doses of glucocorticoids have been primarily linked to pulmonary mucormycosis^14^.

Imaging studies, most notably a CT scan of the sinuses and adjacent tissues, are used to diagnose the illness as they reveal characteristics of edema and destruction of the periorbital bones. Muscle and nerve thickening are observed in cases of orbital involvement. MRI helps in the detection of intradural and intracranial involvement. Cavernous sinus thrombosis manifests as sinus enhancement with a filling defect. Color Doppler imaging helps detect flow abnormality, as seen in internal carotid artery thrombosis, which is a rare complication. Histopathological analysis and direct microscopy are the gold standards for diagnosing mucormycosis. The distinctive presence of broad, irregularly shaped, non-septate, ribbon-like structures with branches frequently emerges at right angles^5^. In addition, fluoroscopy with KOH mount preparations can also be helpful. The fungus can also be cultured in Sabouraud’s dextrose agar media showing cottony growth with black spores. In our case, heterogeneously enhancing soft tissue was present in the left PNS with extension to the left nasal cavity as well as along the left trigeminal nerve and cerebellopontine angle.

Headache, fever, sinusitis, facial edema, orbital apex syndrome, convulsions, altered level of consciousness, coma, stroke, and blindness are among the complications of ROCM. Drooping mouth corners is one of the most notable neurological symptoms as seen in our patient. Though our case was diagnosed on time, there could be delay in diagnosis as culture often yields no growth, and histopathologic identification of an organism with a structure typical of Mucorales may be the only evidence of infection. The identification of the distinctive hyphae in a clinical sample suggests a preliminary diagnosis that necessitates additional investigation.

As part of a multidisciplinary approach, Amphotericin B, the preferred medication for the primary treatment of mucormycosis, Lipid formulations have been shown to have less nephrotoxicity and can be administered for extended periods. However, due to the unavailability of liposomal preparations, non-liposomal formulations were used, which would otherwise have increased nephrotoxicity, which did not develop in our case. Depending on the patient’s response, the course of therapy should be tailored^5^. Posaconazole can be added to the regimen when amphotericin B therapy fails, or the patient is intolerable. Surgical intervention is necessary in addition to medical treatment. Depending on the severity of the disease, debridement, and procedures such as antral wash, lateral rhinotomy, sinusectomy, and even intracranial surgery are performed^5^. The patient should be followed up to assess the recurrence and the success of treatment.

The survival rate is increased by combining medical and surgical treatment^12^. Additionally, it is advised to undergo hyperbaric oxygen therapy with 100% oxygen for 90 to 180 minutes at 2–2.5 atmospheric pressures, with one or two exposures per day^5,12^. Invasive ROCM has a terrible prognosis; if intracerebral and intraorbital complications are present leading to death^8^. Delays in identification and treatment, the inclusion of both sinuses, face necrosis, and other factors have all been linked to poor outcomes^15^.

## Conclusion

Our patient improved gradually and hence was discharged with medications and advice. It is recommended that an early diagnosis of this potentially fatal illness should be made more cautiously in the high-risk groups, including diabetic patients. With prompt treatment, the risk of morbidity and mortality can be significantly decreased.

## Ethical approval

Not applicable.

## Consent

Written informed consent was obtained from the patient for publication of this case report and accompanying images. A copy of written consent is available for review by the Editor-in-Chief of this journal on request.

## Source of funding

Not applicable.

## Author contribution

R.C.S., A.A.: led data collection, contributed in writing the case information. S.G., P.J.: contributed to the process of original draft preparation and introduction and discussion. S.A., T.K.S.: contributed to conceptualization, and discussion. B.B., N.K., S.K.J.: revised it critically for important intellectual content, contributed in review and editing. B.L., R.P., S.K.J.: edited the rough draft into the final manuscript.

## Conflicts of interest disclosure

The authors declare no conflicts of interest.

## Research registration unique identifying number (UIN)

1. Name of the registry: N/A.
2. Unique Identifying number or registration ID: - N/A.
3. Hyperlink to your specific registration (must be publicly accessible and will be checked):- N/A.

## Guarantor

Pinky Jha.

## Data availability statement

Data are available upon reasonable request.

## Provenance and peer review

Not commissioned, externally peer-reviewed.
